# Parent experiences with paediatric allergy pathways in the West Midlands: A qualitative study

**DOI:** 10.1111/cea.13334

**Published:** 2019-02-11

**Authors:** Lavanya Diwakar, Carole Cummins, Scott Hackett, Martyn Rees, Lynette Charles, Caroline Kerrigan, Helen Creed, Tracy Roberts

**Affiliations:** ^1^ Department of Health Economics University of Birmingham Birmingham UK; ^2^ Department of Immunology University Hospital of North Midlands NHS Trust Stoke‐on‐Trent UK; ^3^ Institute of Applied Health Research University of Birmingham Birmingham UK; ^4^ Department of Paediatric Immunodeficiency and Allergy Birmingham Heartlands Hospital NHS Trust Birmingham UK; ^5^ Department of Paediatric Respiratory Medicine Royal Shrewsbury and Telford Hospitals NHS Trust Shrewsbury UK

## Abstract

**Background:**

The prevalence, severity and complexity of allergic diseases have been increasing steadily in the United Kingdom over the last few decades. Primary care physicians are often not adequately trained in allergy management while specialist services for allergy are scarce and heterogeneous. Services, therefore, have been unable to meet the rising demand. This is particularly true for paediatric allergy services in the United Kingdom.

**Objective:**

To understand parent experiences with paediatric allergy pathways in the West Midlands (WM) region of the United Kingdom.

**Methods:**

Parents of children aged between 0 and 16 years from the WM region were recruited opportunistically until thematic saturation was achieved. Eighteen semi‐structured interviews were carried out and transcribed verbatim. Data were analysed on NVivo software using the framework method. Themes were identified from the transcripts as well as from existing literature.

**Results:**

Parents highlighted numerous issues related to allergy services in the region including difficulties with being taken seriously by their physicians, problems with accessing health care and issues with information and the need for additional supportive care for allergies.

**Conclusions and Clinical relevance:**

Primary care for children with allergies in the WM is disparate. Parents experience difficulties in accessing primary and secondary care services and also obtaining timely and appropriate information regarding their child's allergies. Most parents were happy to be reviewed by either specialist nurses or by consultants in the hospital. Improving accessibility and availability of reliable information as well as provision of additional services (such as psychologists and dietetics) were highlighted by parents as being important to allergy services in the region. These findings can help inform future planning and commissioning of allergy services.

## 1 INTRODUCTION

The United Kingdom has among the highest rates of allergy and related diseases in the Western Hemisphere,^1^ and there has been a steady increase in the prevalence, severity and complexity of allergic disease in the last 2‐3 decades.^2^, ^3^, ^4^, ^5^ Some researchers have estimated that a third of all UK adults and a higher proportion of children will be diagnosed with allergies during their lifetime.^6^ Nevertheless, specialist allergy services have remained scarce with very little improvement over the last two decades. There is a well‐documented shortage of trained specialists for allergy, particularly paediatric allergy, in the United Kingdom.^7^, ^8^ Consequently, secondary care services are often provided by clinicians who may not be formally trained in allergy.^9^ Allergy specialist services for children across the country, therefore, vary significantly in terms of both accessibility and quality.^8^, ^10^

Numerous reports have been published in the United Kingdom discussing the shortfall in service provision and the need to reorganize allergy services.^8^, ^9^, ^11^, ^12^, ^13^ More recent publications, including a report from the North East Allergy Project, suggest that a “one size fits all” approach to planning services may not be appropriate.^13^, ^14^ These reports suggest that understanding regional expertise and appraising the needs of the local population are important in planning service reorganization.

We planned a qualitative study aimed at understanding the experiences of individuals accessing allergy services within the West Midlands (WM) region. Since parents access health care on behalf of their children, semi‐structured interviews with parents of children attending two specialist paediatric allergy clinics, that is the Royal Shrewsbury hospital and Birmingham Heartlands hospital, were carried out. These centres were chosen as they are geographically sufficiently separated (about 50 miles) to prevent overlap of services and experiences.

## 2 METHODS

Parents of children aged 0‐16 years with allergies or related conditions attending either of the two specialist clinics during the study period were eligible for inclusion in the study. The study was carried out in two phases: six interviews were carried out in phase 1 (between September 2014 and December 2014) and 12 interviews were carried out in phase 2 (between January 2016 and June 2016) to accommodate parental leave taken by the researcher (LD). Parents aged less than 18 years and those unable to understand or converse in English were excluded.

Parents were recruited by clinicians in the study centres. All interviews were carried out by LD. Interviews were semi‐structured and were based on an interview topic guide. The guide was developed taking into account existing literature regarding experiences of individuals with allergies^15^, ^16^, ^17^, ^18^ and was modified further based on the views expressed by the parents as the interviews progressed (Table 1). Two interim analyses were carried out during the project (December 2014 and then in April 2016) to inform further recruitment and to identify emerging themes. Interviews were carried out until thematic saturation was achieved (ie, no new themes emerged from subsequent interviews).

**Table 1:** Interview topic guide (allergy services related questions covered during interview)

1. Warm‐up Details regarding participant Details regarding child
2. Allergies when diagnosed, by whom, how serious feelings about the diagnosis support with management
3. Primary care ease of access details of the appointment satisfied with the appointment?
4. Referral to secondary care ease of access About the clinic (waiting time, ease of access, distance from home, parking or other issues)
5. Specialist clinic details of the appointment itself Was it useful/ satisfactory?
6. Services for allergy How do you think services for allergy can be improved? (are the services ok the way they are now/ are you happy with them?) impressions regarding primary care for allergy thoughts on the current food labelling systems (where relevant)
7. Closing, thanking and signposting Any other information/points for discussion

Interviews were audio‐recorded with the parent's consent and anonymised before being transcribed verbatim by an external company. Ethical approval was obtained for the study (National Research Ethics Committee reference: 14/NE/1060), and permissions were obtained from the R&D departments of the individual hospitals.

## 3 ANALYSIS

The transcripts were analysed using the NVivo 11 software (QSR international Pty Ltd., Version 11, 2015; http://www.qsrinternational.com/nvivo/support-overview/faqs/how-do-i-cite-nvivo-for-mac,-nvivo-11-forwindows). The framework method as described by Gale et al^19^ was used to code the transcripts and to identify overarching themes. These codes were both inductive (developed de novo from the interviews) and deductive (adapted from the literature).^20^ Interviews were continued until thematic saturation was achieved; that is, no new issues were identified from subsequent interviews. Half of the transcripts were coded by two independent researchers experienced in qualitative research in order to ensure consistency in coding. A final report of the study was sent to participants who expressed an interest in doing so. They were encouraged to contact the researchers if they found any inconsistencies or disagreed with the study findings.

## 4 RESULTS

A total of 22 parents were consented by the centres to take part in the study. Four parents withdrew their participation before the interview due to personal reasons. Of the 18 interviews, 12 were carried out over the telephone and six were conducted in the home of the interviewee (as per interviewee preference). The characteristics of the participants are shown in Table 2.

**Table 2:** Details of parents interviewed

No	ID	Interview^a^	Parent age (y)	Child age (y)	Allergy
1	P 01	Direct	26‐40	1‐5	Eczema, nut
2	P 03	Direct	26‐40	<1	Egg
3	P 06	Direct	26‐40	1‐5	Milk, soya
4	P 07	Telephone	26‐40	1‐5	Eczema, asthma
5	P 08	Direct	26‐40	1‐5	Plum
6	P 09	Direct	26‐40	<1	Multiple food intolerances
7	P 11	Telephone	26‐40	1‐5	Eczema, milk intolerance
8	P 12	Telephone	26‐40	1‐5	Nut allergy
9	P 13	Telephone	26‐40	5‐10	Egg, peanut allergy
10	P 14	Telephone	41‐55	1‐5	Peanut, baked beans, egg
11	P 15	Telephone	41‐55	14	Peanut and macadamia
12	P 16	Telephone	41‐55	<1	Milk, egg and wheat
13	P 17	Telephone	26‐40	1‐5	Peanut
14	P 18	Telephone	41‐55	10‐15	Sesame
15	P 19	Direct	26‐40	1‐5	Egg
16	P 20	Telephone	41‐55	10‐15	Peanut, cat, dog
17	P 21	Telephone	41‐55	>15	Hayfever
18	P 22	Telephone	41‐55	10‐15	Dairy, egg, sesame, nuts

a Direct refers to face‐to‐face interviews.

Parents discussed many issues including the initial symptoms of allergy in their child, previous experiences with allergies, experiences related to allergy services and the effect of the allergies on the child(ren) and the family. This paper will specifically discuss parent experiences with paediatric allergy services.

The following themes related to allergy services emerged from the interviews.

### 4.1 Being taken seriously

Parents whose children had allergies valued having a mutually respectful relationship with their doctor—especially their GP, who was usually their first contact with the healthcare system. They felt thoroughly distressed when they perceived the attitude of the doctor as dismissive.

I don't really feel like my GP has been part of this journey … in fact, the first GP I saw sort of tried to downplay everything… [P14]

I'd come out sometimes and I'd be so frustrated because I felt like, “You weren't listening”. They [the GPs] just wouldn't listen to me. It was as if – you know, “You're just an overreacting mom”. [P6]

On occasion, parents felt that poor advice from their doctor could have harmed their child.

… he [the GP] said, with regards to whether it was nuts or not, he wasn't sure but if we wanted to, we could try it again ‐which was a bit of ropey advice ‐ but we actually did and it did come up again badly… [P17]

the lady doctor that I saw said ‘oh, no, she needs to have at least four or five episodes [before we refer]’ …. but because she [the child] was so bad, I didn't wanna run the risk of giving it to her just for her to have another episode … [P19]

One of the parents had similar issues with a specialist centre whose care, she felt, was more protocol based than individually tailored. She felt helpless and frustrated by some of her experiences.

[The specialist] wouldn't write a letter explaining [my son's] allergies until they made him have a food challenge which infuriated me because he's been in several times with a reaction to milk …. So I had to take him in to witness them – rub milk on, his lips, watch him go into reaction and I'm there thinking, ‘Why the hell have you had to do that to my son, just to write a letter saying, ‘Yeah, he's got a dairy allergy?’ So in that way, I did feel bullied by the clinic sometimes. [P22]

Parents felt some GPs were better than others when it came to managing their child's allergies. They sometimes opted to see different GPs within the same practice, even if this meant waiting a few extra weeks.

…I suppose if I want to be diplomatic about it, there are GPs that I would prefer to see as opposed to one or two others. Some are quite dismissive. [P11]

… because the Doctor I wanted to see that, he was one of the children's Doctors, you know, she's seen right from being little, I normally have to wait, maybe 3, 4 weeks but that's because I want a specific Doctor..because that's who [the child] been seeing all along. [P21]

When the parents felt “listened to” and that their GPs were sympathetic, they were generally accepting of the treatment and management plans, even if the referral was deferred or the treatment was not particularly effective.

He [the GP] just said, if ever you reach the point where it's interfering with life or that there's too much going on…., come back because I can always refer her, and we can just get it sorted out once and for all. [P21]

… yeah, [the GPs] are helpful. They're trying to find the different creams and all the rest of it, but I think it's quite an inexact science in terms of what creams will not react with the child and all the rest of it. [P13]

… I didn't feel at any point that when I went it was like, oh it's not you again! … I'd say definitely with the E45 I thought… well that's not eczema cream, but I can't really fault them for trying the different creams. [P7]

Parents were very critical of doctors who would not acknowledge their lack of knowledge with regards to managing their child.

I think a lot of doctors tend to think that they know everything … and I don't know whether that's because they feel that there's an expectation from their patients that they should know everything. I think with the first GP I saw, there was some degree of making it up as they went along. [P14]

### 4.2 Access to appropriate health care

Parents who accessed emergency services for their child's allergy felt that they were well looked after and then referred to the allergy services as appropriate. Experiences with accessing GP services, however, were more variable. Some GPs had made special provisions for children, and therefore, accessing appointments or advice in these surgeries was reasonably straightforward.

… the receptionist will sometimes say they'll get a doctor to call you back. And you can always speak to a nurse if you don't wanna wait. The nurse will always speak to you, … So in that respect it's, it's okay. [P19]

Others found that getting an appointment with their GP can be very difficult and stressful.

… trying to get an appointment with them was like near on impossible unless you were prepared to kind of go to their sit and wait surgery which is a bit difficult to do if you've got other children with you. [P12]

– it can sometimes take half‐an‐hour of phoning to actually get an appointment in the first place…it's almost like a bit of a lottery. You try and phone to get through and …it'll just come up with the engaged tone. I've actually tried to call about 120 times. And that's no exaggeration. [P11]

Referrals to specialists in the NHS are possible only via the general practitioners or through the emergency department within the hospital. The process was quite straightforward for a few parents.

I saw a GP and explained everything that had happened and she…yeah, just referred us straight on. [P3]

Securing a referral, however, was a stressful process for some parents. They describe their frustration since their child was not being adequately managed in primary care and yet they faced problems with being referred to a specialist. Parents sometimes had to be quite determined to secure a referral.

I'd sat and refused to leave until I had a referral…because a friend of mine actually told me they can't refuse to refer you. So, I said, ‘I'm not leaving now until he gets the referral’. [P6]

I kind of planned my attack. So I specifically made an appointment with one of the female doctors at my surgery…and by the time I'd walked out, I didn't just have a referral; I had an appointment. [P14]

Once a specialist referral was made, most parents expected to wait a while before being able to actually consult a specialist for the first time. This was frustrating for some.

The appointment letter came through pretty quick, … but when you opened the letter all of a sudden we had to wait another three months to be seen. That was very frustrating. [P11]

… the waiting times for the appointments should not be so long, because we've been waiting months for the appointment. [P9]

The reaction to follow‐up care was mixed. While some found them useful, others were critical. They questioned the usefulness of short consultations and also the organization of follow‐up clinics.

[the follow up] was very useful because up until a couple of weeks back [my child] wouldn't entertain eating a nut. So now she is eating other nuts, and she is absolutely fine. [The specialist nurses] at the hospital, you know, they were the catalyst to push her to do that. So from that point of view, it has been very useful. [P15]

… we only really get about ten minutes in the appointment and … so it's not kind of an in‐depth appointment really, it just I feel like there should be a little bit more; … it's the worry of knowing that the reaction is so severe, that anaphylactic shock that's really scary to me so I feel like having a ten minute appointment once a year isn't really enough. [P12]

### 4.3 Clinician seen in hospital

Most (16/18) of the parents interviewed were reviewed in clinic by an allergy specialist nurse. Some had not realized this, although all clinicians normally introduce themselves to the patients ahead of the consultation.

I thought she [the specialist nurse] was a doctor. [P6 and P3]

A majority of the parents were happy to see a nurse:

… I think she's [the nurse specialist] probably had more specialist training in that area than some of the doctors have. She is very experienced and I trust her one hundred per cent. [P20]

…“thorough” was a word that definitely came into my head when I was with her [the specialist nurse]… [P14]

… they tell you at the beginning so initially, I was a bit surprised … but when she then started talking about everything that she'd been doing ‐ I just felt confident that she was the right person to deal with (my child). [P17]

Whereas most parents in our cohort did not express a preference for being seen by doctors in the specialist clinics, some felt that there should be a doctor available as “back‐up” in case of complex conditions where the nurse may be overwhelmed. However, a few parents explicitly preferred seeing a doctor and were disappointed when this was not possible.

… I guess while we've seen the nurses and not the consultant there's perhaps that nagging doubt that if we'd seen the consultant would the advice have been slightly different. [P1]

### 4.4 Support with allergy management

Parents were generally pleased with the care they got from hospitals. Often, the allergy clinics put them in touch with other services such as dermatology or dietetic services so that the parents can be given adequate advice regarding management of their children. Sometimes, such referrals take a long time to materialize and this can cause enormous distress to parents. Sometimes, it was not possible to obtain co‐ordinated care between various specialists, even when this was perhaps warranted.

…(my child) has an eye condition – they call it Giant Capillary Conjunctivitis… but the Allergy Clinic doesn't deal with that kind of allergy … so … for his eyes, he was under another consultant‐but he (the eye consultant) didn't understand allergies. [P22].

Some parents felt that the anxiety and isolation experienced when their children have allergies, particularly after life‐threatening episodes, were not being directly addressed by the healthcare system.

…that asthma [attack] then stopped him – he had to stop all sort of sporty activity for about a month…and then, after that, he struggled to get back into anything sporty and now, … he's not into sport whatsoever. So it did [have] a big knock‐on effect to his life. [P22]

Most parents we talked to seemed to try and “get on with it,” changing their lifestyle as appropriate and self‐managing the stress associated with their child's allergy. Some, however, felt that such stress should be more openly acknowledged and managed by the clinicians.

Is [counselling] offered to children and parents that have had that life‐threatening ordeal which is terrifying?!..Does anyone ever actually follow them up and give them a phone call and say “would you like someone to talk to”? [P20]

### 4.5 Issues with information

As with any other childhood condition, parents greatly valued reliable information regarding their child's allergies. Information was generally obtained from the clinicians, through peer groups or from the Internet. Clinics were sometimes able to provide information that a lot of parents found very useful.

… they did have a Nutritionist who then told me these things that were available on the market, which I would never have known about and … a lot of things we then got on prescription,– I mean we were wheat free as well. [P22]

Since seeing [the specialist nurse], I came home with a cookery book and we've made him chocolate cake that the whole family ate. [P6]

Most parents interviewed had limited information provided by GP or A&E and consequently had to wait until seen by specialist for information regarding their condition.

… we didn't leave the GPs or the hospital (A&E) with anything other than sort of what we'd been told verbally, which was quite limited really. [P3]

On occasion even after being reviewed in secondary care, parents came away confused and feeling that the condition and tests have not been fully explained. This caused a feeling of anxiety and frustration. This quote is from a parent who was seen in a general paediatric clinic (not an allergy clinic).

Yeah, so we went in for these tests and they said, ‘Yes, he's allergic to nuts probably. Bye, bye and good luck. Hope you work it out. That… is kind of how I felt… overall nothing was explained terribly well. [P14]

Most parents obtained information from peers (fellow parents) by having informal conversations with them. This provided them with practical tips and also a sense of “not being alone.”

I go to a group, a little meet up with other mums who are also weaning and trying different diets but you compare notes, you know. [P16]

Lack of such support can lead to a feeling of isolation and increased anxiety.

… primarily, I was the only one [with this problem]. I only knew that other mum for a short time and then she moved away I was the only one whose child was going through this, so [having a local parent support group] might have been quite a good idea. [P14]

Many parents also used the Internet to obtain information regarding allergies. Accessing peer groups on the Internet was particularly popular (eg, Mumsnet, Netmums and Facebook groups) as these seemed to provide emotional as well as practical support to most of the mothers interviewed. The accessibility and anonymity of these forums as well as the empathy that these scenarios generated from other users were particularly comforting to our interviewees.

NetMums is a great one because it's one of them you can put the daftest question regarding a child that if you think it's stupid and there's always a mum that somebody's been through it and, you know, they can give you advice… [P6]

While parents in our cohort appreciated the support provided by the Internet, they recognized that the information obtained was not always accurate and can sometimes be misleading. Parents do prefer obtaining information from reliable websites (eg, NHS choices). However, some found these inadequate and disappointing.

I mean generally the NHS website is brilliant…but when it came to allergies and intolerances it was next to useless really. [P11]

## 5 DISCUSSION

### 5.1 Principal findings

This study shows that parents experience significant barriers while accessing primary and secondary care allergy services for their children. They found getting same‐day appointments with GPs very difficult and describe helplessness and frustration in their inability to access timely medical advice. In addition, GPs were sometimes perceived as being dismissive and not being sufficiently knowledgeable in managing the child's allergy. Referrals to secondary care were also identified as being a problem, and in some cases, the parents had to wait many months and resort to extraordinary practices (eg, refusing to leave the GP surgery) before referrals could be made. Experiences with secondary care were generally positive, although the long waiting times and follow‐up arrangements were criticized by some parents.

Parents appeared to value highly the acknowledgement from others, specifically healthcare providers, that they are indeed justified in their concerns for their child's health. Even in situations where a clinician was not able to offer a diagnosis, parents greatly appreciated being listened to and taken seriously. In addition, they expected to be referred to a specialist if the GP is unable to help. Referral practices, however, were quite variable across GP practices. The reasons for this are not fully understood, but studies suggest that income deprivation, sex and age may influence referral rates for certain conditions.^21^ Even when referrals had been made, the waiting time to see an allergy specialist is usually long. Parents were more frustrated regarding waiting times for secondary care if the child had ongoing problems which were not addressed adequately in primary care.

Access to reliable and appropriate information was an issue which parents found wanting. Many do try to access online information, but are wary about reliability of most websites. Some of the mothers interviewed found peer support websites (such as Netmums and Mumsnet) very useful, mainly for the advice but partly for the empathy that they get from the other users. Parents were unhappy about the lack of information from reliable sources (such as the NHS choices website) regarding allergies. Although there are some good websites offering advice on allergies (RCPCH, Allergy UK, anaphylaxis campaign)—these are clearly not well publicized and GPs, and consequently parents, remain unaware of their existence.

### 5.2 Strengths and limitations of the study

This is the first in‐depth study into parent experiences with allergy services in the United Kingdom. We interviewed parents attending two specialist allergy centres within the WM which allowed us to capture a range of experiences, since these parents had accessed both primary and secondary care services for their children. The findings were fed back to 13 parents who expressed an interest in seeing the final report of the study; none of them disagreed with the contents of the report. Although this study explored services in the WM only, a review of the existing literature suggests that problems with access to allergy care are a UK‐wide issue^9^ and it is conceivable that many of the opinions and experiences highlighted in this study can be generalizable to other parts of the country.

The sample size for this study is much smaller than that of quantitative studies. This is, however, not a limitation as such and is in keeping with other published qualitative research. The depth of information obtained from this study cannot be obtained from a quantitative study (eg, a questionnaire study) using a large patient sample.

One of the limitations of this study could be the limited number of children with specific allergies (see Table 2). However, the purpose of these interviews was not to evaluate the service pathways for specific allergies (eg, food allergy) but to understand parent experiences with general NHS paediatric allergy services in the WM.

Although some of the parents interviewed were non‐Caucasian, we did not specifically attempt to explore the impact of race or ethnicity on parental experiences with allergy services. This may have influenced the findings of the study given that ethnicity is known to impact health‐seeking behaviour and experiences with health care.^22^

Almost all of the children included in this study were reviewed by specialist nurses. Although the experiences in specialist clinic were mainly positive, it is possible that there may have been variations in experiences if more parents had been seen by consultant allergists or immunologists.

Another potential limitation was the under‐representation of fathers in our study. The recruitment process was modified after the interim analyses to attempt to recruit more fathers but this was not possible. Although there may be differences in how parents perceive allergies,^23^ it is not clear whether interviewing more fathers could have changed the findings in this study.

### 5.3 Strengths and limitations in relation to other studies

A previous report into allergy services has reported anecdotes relating to service pathways in the United Kingdom.^12^ These were comments made by callers to a helpline rather than related to in‐depth interviews. Other studies have been published which report children's experiences relating to healthcare services in general^24^; pathways of care related to specific conditions such as diabetes,^25^ children with special health needs^26^; healthcare access for a selected group of people—for example, elders in rural West Virginia.^27^ However, none of these studies have evaluated experiences related to complete service pathways (ie, primary and secondary care, supporting services) for a particular condition.

### 5.4 Implications for clinical services

Allergy services are currently not meeting the requirements of patients or their carers. Given the current constraints on allergy specialist services and on primary care, other ways of improving services should be explored. For example, most mild‐to‐moderate allergies can be successfully self‐managed by individuals (or carers) if good quality information can be made more accessible and this can potentially reduce the burden on primary and secondary care services. Providing web‐based advice or support for patients/carers should also be considered by the NHS.

### 5.5 Future research

Interviewees in this study were limited to those who attended specialist clinics run by personnel specifically trained in allergy. However, the majority of paediatric allergy services in the United Kingdom (and the WM) are provided by non‐specialists (eg, paediatricians or nurses not formally trained in allergy). Interviewing parents who attend non‐specialist secondary care clinics could potentially add to the results of this study and will be considered in the future.

In addition, a majority of allergy sufferers in the United Kingdom do not encounter specialist services. A large proportion is managed in primary care whereas a significant number self‐manage their condition in the community. Their experiences of living with allergy can provide rich data about accessibility of allergy services, educational needs of patients and adequacy of information available to the public regarding allergies.

The themes highlighted in this study were used to design a discrete choice experiment to understand the preferences of parents for paediatric allergy services.

### 5.6 Conclusions

Primary care for children with allergies in the WM is disparate. Parents experience difficulties in accessing primary and secondary care services and also obtaining timely and appropriate information regarding their child's allergies. While some GPs were described as informed and sympathetic others were found to lack knowledge regarding allergies, causing parents a lot of distress. Access to reliable information regarding allergies was also difficult for most parents. Experiences with secondary care were mostly positive although there were some issues with prolonged waiting times, follow‐up clinics and also with accessing input from other specialists, where needed.

### 5.7 Reflection

As with any qualitative review, the epistemological viewpoint of the researcher (LD) may have affected the findings in this study.^28^ This is especially important to acknowledge given that LD had many things in common with the interviewees (who were mostly working women with young children). In addition, LD is a secondary care clinician with expertise in allergies and her knowledge in this disease area could have influenced this project.

## CONFLICT OF INTEREST

The authors declare no conflict of interest.
